# Spiking neural network connectivity and its potential for temporal sensory processing and variable binding

**DOI:** 10.3389/fncom.2013.00182

**Published:** 2013-12-19

**Authors:** Julie Wall, Cornelius Glackin

**Affiliations:** ^1^Multimedia and Vision Research Group, School of Electronic Engineering and Computer Science, Queen Mary, University of LondonLondon, UK; ^2^Adaptive Systems Research Group, Department of Computer Science, University of HertfordshireHatfield, Hertfordshire, UK

**Keywords:** cell assembly, spiking neural network, spike timing, biological neurons, learning, connectivity, sensory processing

The most biologically-inspired artificial neurons are those of the third generation, and are termed spiking neurons, as individual pulses or spikes are the means by which stimuli are communicated. In essence, a spike is a short-term change in electrical potential and is the basis of communication between biological neurons. Unlike previous generations of artificial neurons, spiking neurons operate in the temporal domain, and exploit time as a resource in their computation. In 1952, Alan Lloyd Hodgkin and Andrew Huxley produced the first model of a spiking neuron; their model describes the complex electro-chemical process that enables spikes to propagate through, and hence be communicated by, spiking neurons. Since this time, improvements in experimental procedures in neurobiology, particularly with *in vivo* experiments, have provided an increasingly more complex understanding of biological neurons. For example, it is now well-understood that the propagation of spikes between neurons requires neurotransmitter, which is typically of limited supply. When the supply is exhausted neurons become unresponsive. The morphology of neurons, number of receptor sites, amongst many other factors, means that neurons consume the supply of neurotransmitter at different rates. This in turn produces variations over time in the responsiveness of neurons, yielding various computational capabilities. Such improvements in the understanding of the biological neuron have culminated in a wide range of different neuron models, ranging from the computationally efficient to the biologically realistic. These models enable the modeling of neural circuits found in the brain.

In recent years, much of the focus in neuron modeling has moved to the study of the connectivity of spiking neural networks. Spiking neural networks provide a vehicle to understand from a computational perspective, aspects of the brain's neural circuitry. This understanding can then be used to tackle some of the historically intractable issues with artificial neurons, such as scalability and lack of variable binding. Current knowledge of feed-forward, lateral, and recurrent connectivity of spiking neurons, and the interplay between excitatory and inhibitory neurons is beginning to shed light on these issues, by improved understanding of the temporal processing capabilities and synchronous behavior of biological neurons. This research topic spans current research on neuron models to spiking neural networks and their application to interesting and current computational problems. The research papers submitted to this topic can be categorized into the following major areas of more efficient neuron modeling; lateral and recurrent spiking neural network connectivity; exploitation of biological neural circuitry by means of spiking neural networks; optimization of spiking neural networks; and spiking neural networks for sensory processing.

Moujahid and d'Anjou (2012) stimulate the giant squid axon with simulated spikes to develop some new insights into the development of more relevant models of biological neurons. They observed that temperature mediates the efficiency of action potentials by reducing the overlap between sodium and potassium currents in the ion exchange and subsequent energy consumption. The original research article by Dockendorf and Srinivasa (2013) falls into the area of lateral and recurrent spiking neural network connectivity. It presents a recurrent spiking model capable of learning episodes featuring missing and noisy data. The presented topology provides a means of recalling previously encoded patterns where inhibition is of the high frequency variety aiming to promote stability of the network. Kaplan et al. (2013) also investigated the use of recurrent spiking connectivity in their work on motion-based prediction and the issue of missing data. Here they address how anisotropic connectivity patterns that consider the tuning properties of neurons efficiently predict the trajectory of a disappearing moving stimulus. They demonstrate and test this by simulating the network response in a moving-dot blanking experiment.

Garrido et al. (2013) investigate how systematic modifications of synaptic weights can exert close control over the timing of spike transmissions. They demonstrate this using a network of leaky integrate-and-fire spiking neurons to simulate cells of the cerebellar granular layer. Börgers and Walker (2013) investigate simulations of excitatory pyramidal cells and inhibitory interneurons which interact and exhibit gamma rhythms in the hippocampus and neocortex. They focus on how inhibitory interneurons maintain synchrony using gap junctions. Similarly, Ponulak and Hopfield (2013) also take inspiration from the neural structure of the hippocampus to hypothesize about the problem of spatial navigation. Their topology encodes the spatial environment through an exploratory phase which utilizes “place” cells to reflect all possible trajectory boundaries and environmental constraints. Subsequently, a wave propagation process maps the trajectory between the target or multiple targets and the current location by altering the synaptic connectivity of the aforementioned “place” cells in a single pass. A novel viewpoint of the state-of-the-art for the exploitation of biological neural circuitry by means of spiking neural networks is provided by Aimone and Weick (2013). In their paper, a thorough and comprehensive review of modeling cortical damage due to stroke is provided. They argue that a theoretical understanding of the damaged cortical area post-disease is vital while taking into account current thinking of models for adult neurogenesis.

One of the issues with modeling large-scale spiking neural networks is the lack of tools to analyse such a large parameter space, as Buice and Chow (2013) discuss in their hypothesis and theory article. They propose a possible approach which combines mean field theory with information about spiking correlations; thus reducing the complexity to that of a more comprehensible rate-like description. Demonstrations of spiking neural networks for sensory processing include the work of Srinivasa and Jiang (2013). Their research consists of the development of spiking neuron models, initially assembled into an unstructured map topology. The authors show how the combination of self-organized and STDP-based continuous learning can provide the initial formation and on-going maintenance of orientation and ocular dominance maps of the kind commonly found in the visual cortex.

It is clear that research on spiking neural networks has expanded beyond computational models of individual neurons and now encompasses large-scale networks which aim to model the behavior of whole neural regions. This has resulted in a diverse and exciting field of research with many perspectives and a multitude of potential applications.
